# Regulation of p27 and Cdk2 Expression in Different Adipose Tissue Depots in Aging and Obesity

**DOI:** 10.3390/ijms222111745

**Published:** 2021-10-29

**Authors:** Ignacio Colón-Mesa, Marta Fernández-Galilea, Neira Sáinz, Marta Lopez-Yus, Jose M. Artigas, José Miguel Arbonés-Mainar, Elisa Félix-Soriano, Xavier Escoté, María Jesús Moreno-Aliaga

**Affiliations:** 1 Center for Nutrition Research and Department of Nutrition, Food Science and Physiology, University of Navarra, 31008 Pamplona, Spain; icolon@alumni.unav.es (I.C.-M.); mfgalilea@unav.es (M.F.-G.); nsainz@unav.es (N.S.); efelix@alumni.unav.es (E.F.-S.); mjmoreno@unav.es (M.J.M.-A.); 2 Navarra Institute for Health Research (IdiSNA), 31008 Pamplona, Spain; 3 Adipocyte and Fat Biology Laboratory (AdipoFat), Unidad de Investigación Traslacional, Instituto Aragonés de Ciencias de la Salud (IACS), Instituto de Investigación Sanitaria (IIS) Aragón, Hospital Universitario Miguel Servet, 50009 Zaragoza, Spain; martalyus@gmail.com (M.L.-Y.); jmarbones.iacs@aragon.es (J.M.A.-M.); 4 Radiology Department, Hospital Universitario Miguel Servet, 50009 Zaragoza, Spain; jmartigasm@gmail.com; 5 CIBERobn Physiopathology of Obesity and Nutrition, Carlos III Health Institute, 28029 Madrid, Spain; 6 Department of Biochemistry and Biotechnology, Universitat Rovira i Virgili, Campus Sescelades, 43007 Tarragona, Spain; 7 Eurecat, Technology Centre of Catalunya, Nutrition and Health Unit, 43204 Reus, Spain

**Keywords:** aging, obesity, cell cycle, p27, CDK2, cyclins, adipose tissue

## Abstract

Aging usually comes associated with increased visceral fat accumulation, reaching even an obesity state, and favoring its associated comorbidities. One of the processes involved in aging is cellular senescence, which is highly dependent on the activity of the regulators of the cell cycle. The aim of this study was to analyze the changes in the expression of *p27* and *cdk2* in different adipose tissue depots during aging, as well as their regulation by obesity in mice. Changes in the expression of *p27* and *CDK2* in visceral and subcutaneous white adipose tissue (WAT) biopsies were also analyzed in a human cohort of obesity and type 2 diabetes. *p27*, but not *cdk2*, exhibits a lower expression in subcutaneous than in visceral WAT in mice and humans. *p27* is drastically downregulated by aging in subcutaneous WAT (scWAT), but not in gonadal WAT, of female mice. Obesity upregulates *p27* and *cdk2* expression in scWAT, but not in other fat depots of aged mice. In humans, a significant upregulation of *p27* was observed in visceral WAT of subjects with obesity. Taken together, these results show a differential adipose depot-dependent regulation of *p27* and *cdk2* in aging and obesity, suggesting that p27 and cdk2 could contribute to the adipose-tissue depot’s metabolic differences. Further studies are necessary to fully corroborate this hypothesis.

## 1. Introduction

Aging is a complex process due to the interaction of genetic, epigenetic, environmental and stochastic factors throughout life, which usually comes together with an accumulation of visceral fat, causing obesity [1,2,3]. It has been estimated that approximately 37% of the world population over 60 years old suffers from obesity [4,5]. With age, lifestyle is also modified, becoming more sedentary and contributing this way to a reduction of energy expenditure [1], favoring the development of obesity, increasing the risk of suffering cardiovascular disorders, diabetes, cerebrovascular diseases, several types of cancer, declined physical function, and loss of independence [6,7,8]. With aging, the distribution of body fat changes from subcutaneous to visceral, causing an increase of abdominal white adipose tissue (WAT). The subcutaneous WAT (scWAT) is found under the skin, where it functions as a protective and isolating barrier to avoid heat loss, whereas visceral WAT (vWAT) is localized surrounding vital organs in the peritoneum and rib cage [9]. Despite both being white fat, they are heterogeneous and their implication in the metabolic complications caused by obesity is different. The accumulation of vWAT facilitates the development of obesity-associated comorbidities, while the accumulation of scWAT, especially in the gluteofemoral area, seems to have beneficial effects against metabolic syndrome [9,10]. In obesity, the expansion of adipose tissue is due to an increase in size (hypertrophy) and in the adipocyte number (hyperplasia) [11]. Additionally, triglycerides can also ectopically deposit in the liver, muscle and heart. This ectopic accumulation facilitates alterations in organs where fat is accumulated, causing lipotoxicity and fostering the development of pathologies such as insulin resistance and type 2 diabetes mellitus (DM), among others [1]. Therefore, adipose tissue dysfunction and its redistribution, especially in obesity, can favor the aging process [1,8]. Additionally, they have both been similarly defined as a low-grade inflammation state, which promotes the development of obesity- and age-related diseases [8,12,13]. Conversely, brown adipose tissue (BAT) is a thermogenic tissue, and the increase of its thermogenic activity contributes to the rise of energy expenditure. In humans, BAT is essential in the first stages of life to maintain body temperature [14]. Apart from these functions, BAT is also involved in glucose homeostasis, triglyceride clearance and insulin sensitivity [15,16]. While WAT mass expands in obesity, BAT activity is inversely associated with body mass index (BMI) because activity is lower in obese and old subjects [17,18].

Adipose tissue alterations manifest not only due to aging but also due to a cessation of cell division, which is known as cellular senescence [19,20]. Cellular division follows a highly coordinated process mainly regulated by cyclin dependent kinases (CDKs). CDKs are kinases that activity depends on regulatory subunits, cyclins, which have no enzymatic activity by themselves. Instead, they associate to a CDK, forming active complexes to carry out their function [21]. The CDK–cyclin complexes are inhibited by specific inhibitors (CDKIs) [22], which are classified into two families: the INK4 family, which specifically inhibits CDK4/6 complexes and the CIP/KIP family (p21, p27 and p57), which are able to inhibit a wide range of CDK–cyclin complexes [23]. Specifically, p27 inhibits complexes of CDK2 and cyclin A (ccna) or E (ccne) and has a critical function in the negative regulation of cellular growth [24]. Apart from their functions in cell cycle regulation, several studies have revealed that some CDKs may play an important role in metabolism [21] such as CDK4, CDK5 or CDK7 [25,26,27,28]. Furthermore, adipose tissue-related functions have also been attributed to some cell cycle regulators. In this sense, it was reported that p27 deficient mice have an altered adipogenesis and a greater adipose mass [29,30]. Additionally, it was shown that the phosphorylation by CDK2 of the CCAAT/enhancer binding protein-β (C/EBPβ) was necessary for adipocyte differentiation to occur [31,32]. Several studies have pointed out a possible role of p27 and CDK2 in adipose tissue development and metabolism as well as in aging. Thus, we aimed to analyze the regulation of the gene expression of *cdk2*, *p27*, *ccna* and *ccne* in different adipose tissue depots during aging, as well as in obesity in mouse models. Additionally, the expression of both *CDK2* and *p27* was also analyzed in vWAT and scWAT depots from normal-weight, overweight and obese subjects with or without type 2 DM.

## 2. Results

### 2.1. Effects of Aging and Long-Term High Fat Diet (HFD) on Body Fatness and Weights of Different Fat Depots in Female Mice

**Figure 1:**
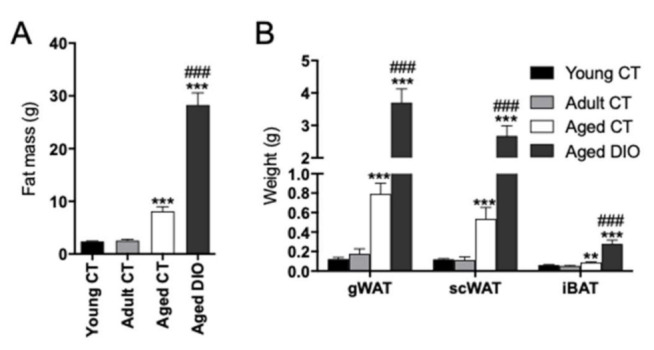
Changes in adipose tissue mass induced by aging and obesity in female mice. (**A**) Whole body fat mass measured by magnetic resonance technology in non-fasted mice fed a control diet (CT) at 2 (young CT), 6 (adult CT) and 18 (aged CT) months of age or aged under a HFD up to 18-month-old (aged DIO); (**B**): weights of the different adipose tissue depots in CT mice (young, adult, and aged) and in aged DIO mice after an overnight fasting. Data are expressed as mean ± SEM. ** *p* < 0.01, *** *p* < 0.001 vs. young CT mice; ^###^ *p* < 0.001 vs. aged CT mice *(n* = 7–9).

The experimental groups used in this study represent different life stages. The 2-month-old group would correspond to a young human age, the 6-month-old groups to adulthood and the 18-month-old groups to a late life stage in humans [33]. Total body fat mass was analyzed by magnetic resonance technology. As expected, aging (18-month-old mice vs. 2- and 6-month-old mice) was accompanied by a significant increase of fat mass even in mice fed with a control diet (aged CT) (Figure 1A). Aging-induced fat mass accumulation was severely potentiated in diet-induced obese mice (aged DIO), chronically fed with a HFD. The weights of the different adipose tissue depots, gonadal adipose tissue (gWAT), scWAT and interscapular BAT (iBAT) were significantly increased in aged CT mice, especially in those grown under obesogenic conditions (aged DIO). Nevertheless, fat accumulation was more pronounced in gWAT and scWAT than in iBAT of aged DIO mice (Figure 1B).

### 2.2. Differential Expression of p27/cdk2/ccna/ccne between Adipose Tissue Depots in Female Mice

**Figure 2:**
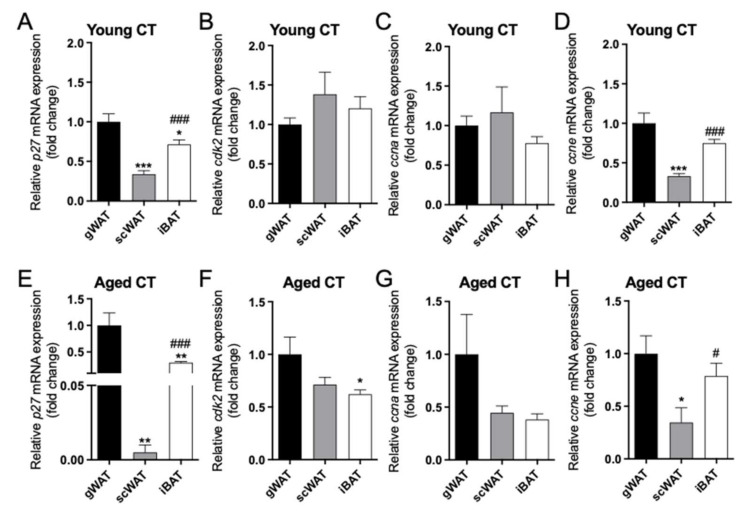
Differential adipose depot expression of *p27* (**A**,**E**), *cdk2* (**B**,**F**), *ccna* (**C**,**G**), *ccne* (**D**,**H**)*,* between gWAT, scWAT and iBAT in young CT mice (2-month-old) and aged CT female mice (18-month-old). Data (mean ± SEM) are expressed as fold change of gWAT, considered as 1. * *p* < 0.05, ** *p* < 0.01, *** *p* < 0.001 vs. gWAT; ^#^ *p* < 0.05, ^###^ *p* < 0.001 vs. scWAT (*n* = 5–8).

We next aimed to study the potential differences in the mRNA expression pattern of several cell cycle regulators (*cdk2*, *p27*, *ccne*, *ccna*) among the three different adipose tissue depots (gWAT vs. scWAT vs. iBAT) in young CT mice. Regarding *p27* (Figure 2A), the highest expression levels were found in gWAT, as compared to iBAT and, above all, to scWAT. Contrariwise, *cdk2* expression (Figure 2B) and *ccna* expression (Figure 2C) showed similar expression levels in the three fat depots. In contrast, *ccne* mRNA presented a pattern of changes similar to those observed for *p27*, showing a decreased expression in scWAT compared to iBAT or gWAT (Figure 2D).

In aged CT mice, the expression of *p27* followed a similar trend as that observed in the young CT mice, although the decrease observed in scWAT *p27* mRNA was more pronounced (Figure 2E). In contrast, the expression of *cdk2* in iBAT was reduced compared to gWAT (Figure 2F), which differs with the lack of changes observed in young CT mice. Regarding cyclins, *ccna* tended to be decreased in scWAT and iBAT as compared to gWAT (Figure 2G), and *ccne* showed a decreased expression in scWAT vs. gWAT and iBAT. (Figure 2H).

These data suggest a differential expression of *p27* and *ccne* between vWAT and scWAT that could be more pronounced during aging for *p27*.

### 2.3. Changes in the Expression of p27/cdk2/ccna/ccne within each Adipose Tissue Depot during Aging in Female Mice

**Figure 3:**
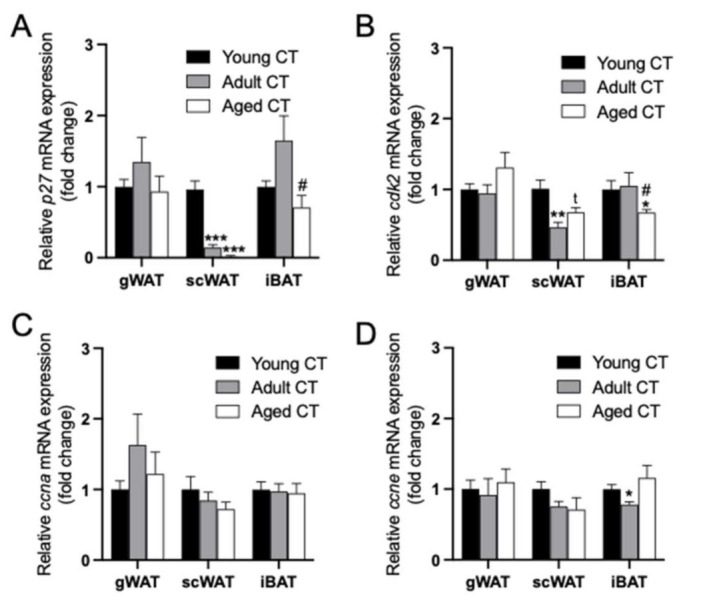
Changes induced by aging on the mRNA expression of *p27* (**A**); *cdk2* (**B**); *ccna* (**C**); *ccne* (**D**) within gWAT, scWAT and iBAT from young, adult and aged (2-, 6- and 18-month-old) CT female mice. Data (mean ± SEM) are expressed as fold change of Young CT, considered as 1. * *p* < 0.05, ** *p* < 0.01, *** *p* < 0.001 vs. young CT; ^#^ *p* < 0.05 vs. adult CT; ^t^ *p* = 0.07 vs. Young CT (*n* = 5–8).

To better characterize the changes induced by aging in the mRNA expression of these cell cycle regulators, gene expression levels of *p27*/*cdk2*/*ccna*/*ccne* within each adipose tissue levels at young, adult and aged mice were analyzed (Figure 3). Regarding the *p27* expression in the aging process, no changes were observed in the gWAT depot (Figure 3A). In contrast, *p27* expression in scWAT was drastically (*p* < 0.001) decreased in adult (6-month-old) and in aged (18-month-old) CT mice in comparison to young CT (2-month-old) mice. In iBAT, adult CT mice showed a slight non-significant increase in the *p27* expression compared to young CT mice that was totally reversed in aged CT mice (Figure 3A).

In the aging process, no differential expression of *cdk2* was observed in gWAT depot. However, in scWAT, a reduced *cdk2* expression was observed in adult and aged CT mice in comparison to young CT mice. Finally, the iBAT *cdk2* expression was significantly reduced in the older animals (Figure 3B). Concerning both cyclins, no changes were observed with aging within any adipose tissue depot, except for a decreased expression of *ccne* in iBAT in adult CT mice group (Figure 3C,D).

As the expression of *p27* and *cdk2* was affected by aging in some fat depots, additional cell cycle regulators were similarly analyzed. In contrast to what was observed for *p27*, *p21* mRNA levels were significantly increased in scWAT when compared to gWAT, and the expression was even higher in iBAT compared to that in scWAT and gWAT (Supplemental Figure S1A). Moreover, aging induced a differential regulation on the expression of *p21* characterized by an increase in gWAT, no changes in scWAT, and a marked decrease in iBAT of aged mice (Supplemental Figure S1C). In contrast to *p21*, *p57* showed a lower expression in scWAT than in gWAT, and again iBAT showed the highest expression of *p57* (Supplemental Figure S1B). Similar to that observed for *p21*, aging induced an upregulation of *p57* in gWAT and a downregulation in iBAT (Supplemental Figure S1D). 

### 2.4. Effects of Diet-Induced Obesity on the Expression of p27/cdk2/ccna/ccne within each Adipose Tissue Depot in Aged Female Mice

**Figure 4:**
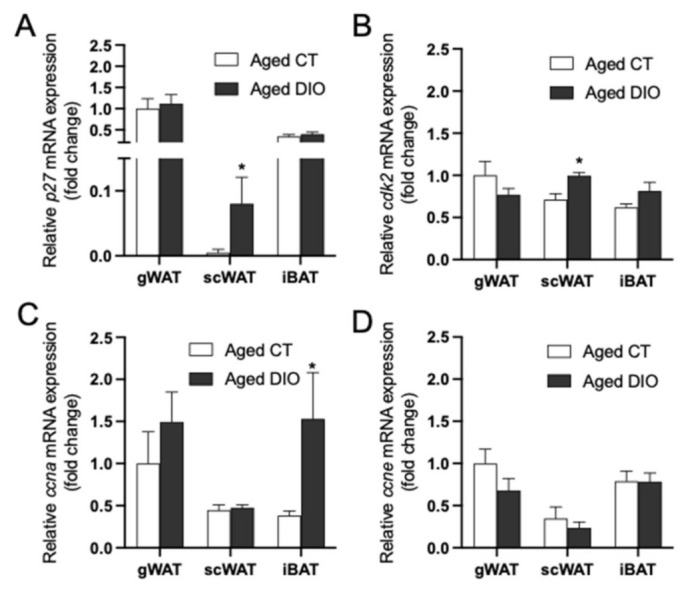
Effect of diet-induced obesity (DIO) on the expression of *p27* (**A**), *cdk2* (**B**), *ccna* (**C**), and *ccne* (**D**) in gWAT, scWAT and iBAT from aged (18-month-old) female mice. Data (mean ± SEM) are expressed as fold change of Aged CT gWAT considered as 1. * *p* < 0.05 vs. aged CT mice (*n* = 5–9).

To further study the effect that obesity may have on the expression of the cell cycle regulators under study, a group of mice were fed with a HFD from 2 to 18 months of age (aged DIO mice) and compared with aged CT mice. The expression of *p27* (Figure 4A) and *cdk2* (Figure 4B) was significantly increased in scWAT of DIO mice as compared to aged CT mice, but no differences on the expression of *p27* or *cdk2* were found in gWAT or in iBAT. This effect was also found in adult (5-month-old) male DIO mice, observing an upregulation of *p27* (1.00 ± 0.22 vs. 154.40 ± 26.73; *p* < 0.001, for CT vs. DIO) and *cdk2* (1.00 ± 0.16 vs. 1.59 ± 0.08; *p* < 0.05, for CT vs. DIO) mRNA levels exclusively in scWAT. In the case of *ccna* and *ccne* expression, no differences were observed in the context of obesity, except for an increase in *ccna* mRNA levels in iBAT of aged DIO female mice (Figure 4C,D, respectively).

To further study the role of *p27* and *cdk2* in obesity, correlation analyses among gene expression levels and the adipose tissue weights were performed for each depot (Table 1). A positive association between *ccna* and gWAT mass was observed and higher levels of *p27* and *cdk2* were associated to a larger scWAT depot size. Increased levels of *cdk2* were also associated with elevated levels of *leptin* (*Lep*) in scWAT (Supplemental Table S1). Additionally, increased *cdk2* and *ccna* mRNA levels were associated to heavier iBAT depot.

Concerning the changes induced by obesity in other cell cycle regulators analyzed, no significant changes on *p21* or *p57* gene expression were observed in aged DIO mice (Supplemental Figure S1E,F).

### 2.5. Expression of p27 and CDK2 mRNA in vWAT and scWAT of Men and Women: Regulation by Obesity and Type 2 DM and Correlation Analyses with Age, BMI, vWAT and scWAT Area, Serum Glucose and Triglycerides

The upregulation observed on *p27* and *cdk2* in scWAT of DIO mice moved us to analyze if this phenomenon was also observed in human WAT. The mRNA expression of *p27* and *CDK2* was analyzed in scWAT and vWAT biopsies from subjects (men and women) with normal-weight, overweight, obesity and obesity with type 2 DM. Table 2 shows the main characteristics of the subjects included in the study. As expected, the obesity + DM group presented higher fasting glycemia, insulin levels and glycated hemoglobin (HbA1c) levels when compared to every other group. Curiously, this group showed reduced levels of cholesterol and LDL-cholesterol compared to the normal-weight group.

**Figure 5:**
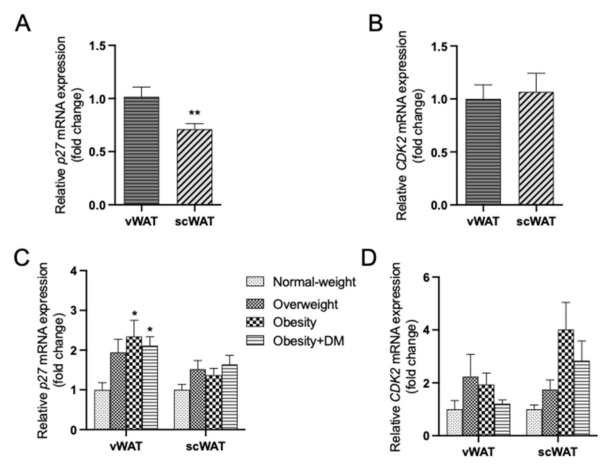
(**A**,**B**): Differential mRNA expression of *p27* and *CDK2* in human vWAT vs. scWAT. Data (mean ± SEM) are expressed as fold change of vWAT considered as 1. ** *p* < 0.01 vs. vWAT. (**C**,**D**). Effects of overweight and obesity with or without type 2 DM on the mRNA expression of *p27* (**C**) and *CDK2* (**D**) in human vWAT and scWAT. Data (mean ± SEM) are expressed as fold change of normal-weight group, considered as 1. * *p* < 0.05, vs. normal-weight.

First, the expression of both *p27* and *CDK2* was compared between both fat depots, showing a lower expression of *p27* in scWAT when compared to vWAT (Figure 5A,B). The expression of *p27* (Figure 5C) was significantly increased in vWAT of the obese groups when compared to the normal-weight subjects. A similar trend was observed for *p27* mRNA levels in scWAT, although without reaching statistical significance. (Figure 5C). A similar trend was observed when the cohort was separated in men and women (Supplemental Figure S2). Regarding *p27*, men and women had comparable pattern of increase in the expression levels with obesity especially in vWAT (Supplemental Figure S2A,C). When studying the expression of *CDK2*, no significant changes were found, although a trend to increase was observed in scWAT from overweight/obese groups (Figure 5D). A similar pattern was observed when analyzing *CDK2* changes comparing men and women (Supplemental Figure S2B,D). As little differences were observed regarding *ccna* and *ccne* on the mouse model, their expression was not analyzed on human subjects samples.

The potential association between *p27* and *CDK2* mRNA WAT expression with obesity and circulating biomarkers of glucose and lipid metabolism was next investigated. Correlation analyses (Table 3) revealed a significant positive association between the expression levels of *p27* in scWAT and the size of this fat depot. Higher *CDK2* expression in scWAT was also associated with a bigger scWAT depot area. In contrast to that observed for the scWAT depot, no associations were found between the expression of *p27* or *CDK2* in vWAT and the size of the depot.

These correlations were also analyzed when the cohort was split in men and women, observing the positive correlation between *CDK2* mRNA levels in scWAT with BMI and triglycerides in men, but not in women. Additionally, *p27* and *CDK2* mRNA expression in scWAT tended to be associated with larger scWAT depot in men, but this tendency was not as pronounced in women. However, in women, *CDK2* mRNA levels in vWAT were correlated to scWAT area (Supplemental Tables S2 and S3).

## 3. Discussion

In this study, we characterized the differential expression of several cell cycle regulators (*p27, cdk2, ccna* and *ccne*) between the different fat depots, as well as their regulation during aging and obesity and their potential implications in these physiological/pathophysiological situations. It is well known that a chronic consumption of HFD produces adipocyte hyperplasia and hypertrophy, which increases simultaneously the degree of inflammation and the chance of developing obesity-associated comorbidities [34,35]. Previous studies have proven the implication of some cyclins and CDKs in adipogenesis regulating processes [36,37]. In this sense, various CDKIs, including p21 and p27, are related with cell proliferation and differentiation in adipose tissue, which could be also associated with adipocyte hyperplasia [36,38]. Moreover, it has been described that during the aging process, a relevant redistribution of body fat occurs, characterized by a higher accumulation of vWAT and a decrease of scWAT (especially on the lower extremities) caused by the progressively diminished lipid storing capacity [39,40]. However, the underlying mechanisms are yet to be described. Moreover, cell cycle regulators are closely related to cellular senescence [41,42], which is one of the various processes involved in aging [43]. 

First, the differences on the expression of *p27, cdk2* and related cyclins in study among the different adipose tissue depots from 2- and 18-month-old mice pointed to an adipose tissue depot-dependent regulation for *p27*. One of the most consistent findings is the observation that *p27* mRNA expression is lower in scWAT as compared to vWAT. This was observed when comparing gWAT and scWAT of both young and aged CT female mice. A similar finding was detected in 5-month-old male mice, which also exhibited lower *p27* expression in scWAT vs. epididymal fat (0.003 ± 0.007 vs. 1.00 ± 0.08; *p* < 0.001, respectively). More importantly, this observation was also found in scWAT from humans. These findings suggest that the lower expression of *p27* in scWAT vs. vWAT could partially account for the metabolic differences between both white fat depots. In this context, it has been described that subcutaneous preadipocytes present a higher proliferation rate [10,44], which could be in part explained by the lower expression of *p27* within this depot. Studies in *p27* knockout mice have shown that the lack of p27 leads to increased fat pad size because of adipocyte hyperplasia [29]. This suggests that the lower expression of *p27* in scWAT as compared to vWAT could be a mechanism for favoring fat accumulation within the subcutaneous depot more than in the visceral fat pads. Furthermore, *ccne* was also reduced in scWAT vs. gWAT and iBAT of young and aged CT mice.

Aging also induced relevant depot-dependent changes in *p27*. For instance, a dramatic decrease of the expression of *p27* in scWAT was also observed due to aging. These changes seem to be depot-specific since they were not observed in gWAT. A more moderate reduction in *cdk2* mRNA levels was also observed in scWAT but not in gWAT of aged mice. This suggests a possible association between p27 and cdk2 and the multiple alterations occurring in scWAT with aging [40]. Our data suggest that the decrease in *p27* expression observed in scWAT of aged CT mice could also contribute to the increased size of this fat pad in old mice as compared to those of young and adult mice. Some studies have suggested that p27 could play a role in cellular senescence during aging [45]. To the best of our knowledge, our study is the first characterizing the changes in *p27* and *cdk2* during aging in adipose tissue depots. Our current data suggest that neither *p27* nor *cdk2* gene expression levels could be considered as markers for the increased cellular senescence during aging in adipose tissue. However, it should be considered that the subcellular location of p27 is critical for its function. It has been proposed that nuclear p27 promotes quiescence, apoptosis, and senescence, while cytoplasmic p27 enhances cell survival and autophagy. Aging has been related with a greater expression of nuclear p27 in several cell types [46]. Therefore, it would be of interest for future studies to characterize at protein level the changes in cellular location of p27 in adipose tissue during aging. 

Conversely, unlike *p27,* the senescence markers *p21* and *p57* [47,48] were not significantly modified in scWAT of aged mice. However, a significant increase of both was observed in the gWAT of aged mice, suggesting a depot-specific role/regulation of these cell cycle regulators during the aging process. The expression of *p21* was found to be increased in the gWAT of old female mice [49] and to be involved on later stages of differentiation and on adipocyte hypertrophy [50].

Our data have also revealed that the long-term high fat feeding during the process of aging provoked a significant increase of the expression of *p27* and *cdk2* exclusively in scWAT, suggesting a likely involvement in the regulation of the expandability of this depot in obesity [31,51]. No changes were observed in the gWAT for neither *cdk2* nor *p27* expression. This result suggests a possible implication of *p27* and *cdk2* on the expansion of scWAT and not gWAT in obesity, which could be due to the physiological difference of both depots [52]. The higher expression of *p27* in scWAT of aged obese mice might make difficult the hyperplasia and fat accumulation in this depot, favoring unhealthy fat accumulation in vWAT. In agreement with our data, expression of *p27* was reported no to suffer changes due to obesity in gWAT; however, at the protein level, an underexpression in 30-week-old obese mice, fed with HFD for 26 weeks, was observed [53]. In contrast to what was observed for *p27* mRNA, our data revealed no changes on the expression of *p57* nor *p21* in the aged DIO group in any of the depots. However, previous studies have suggested a link between p21 and obesity by promoting adipose tissue expansion during high fat feeding [50]. Concerning p57, a study has shown that the increase of the expression of *p57* during development protects against age and diet-induced obesity [54].

Additionally, it is well known that the activity of BAT is highly affected by aging, being almost non-existent in older people [55,56,57] and in obesity [58,59]. This lower activity of BAT is related to a higher susceptibility of suffering obesity and type 2 DM and an increase of BAT activity has been suggested as a possible therapeutic strategy against obesity due to its thermogenic function [58,60]. We have recently reported a reduced iBAT activity in aged CT mice that was aggravated in aged DIO mice [61]. The various factors contributing to the loss of BAT with age have not been yet established [62]. Our current data show a decrease of the expression of *cdk2* in aged BAT, highlighting the importance of studying the potential role of p27 and cdk2 in the lowering of BAT activity occurring with aging [57]. In this way, a previous study has shown that transgenic mice overexpressing p27 specifically in adipocytes, did not apparently modify WAT, but caused a marked reduction in the amount of BAT, which exhibited lower content of uncoupling protein 1 [63]. We have also found a relevant decrease of *p21* and *p57* in iBAT of aged CT mice as compared with young CT mice, suggesting a potential role of these cell cycle inhibitors in iBAT affectation during aging. Our current data also revealed a marked increase in *ccna* mRNA levels in iBAT of aged DIO mice as compared to aged CT mice. Moreover, correlation analyses have shown that higher levels of expression of cell cycle regulators such as *cdk2* and *ccna* are associated with bigger iBAT size in aged CT and DIO female mice, suggesting a possible involvement in brown adipocyte hypertrophy and/or hyperplasia.

However, no relevant changes were observed for *ccna* or *ccne* mRNA levels in gWAT or scWAT during aging or in response to HFD. This might apparently contrast with a previous study showing an increase of cyclin E in mouse preadipocytes after 6–10 weeks of HFD [64]. Therefore, it is important to perform future studies to characterize the role of p27 and cdk2 in the development and function of the different types of adipose tissue. One of the limitations of the present study is that it is not possible to discern whether the alterations of the expression of *p27*, *cdk2 or ccna/ccne* in adipose tissue during aging or obesity are a consequence or cause of these processes. Additionally, although good correlations between mRNA and protein levels for p27 [65,66] and for cyclins are usually described [67], data obtained from the present study is restricted to the mRNA expression. Consequently, to obtain a more complete picture of the role of p27-cdk2 in aging and obesity, the protein levels in adipose tissue should be analyzed in further studies. 

If the regulation of cdk2 and p27 during aging or in obesity is relevant for the development and function of adipose tissue depots, these should be evolutionary conserved between different species [68]. For that reason, the expression of *p27* and *CDK2* mRNA was characterized in vWAT and scWAT of apparently healthy overweight/obese subjects or in obese-diabetic subjects and compared to normal-weight healthy women/men. The results showed a significant increase of the expression of *p27* in the vWAT of subjects with obesity and obesity with type 2 DM. However, this increased expression of *p27* was not observed in the gWAT of mice. This could be attributed to several factors such as the differential regulation in both species [69], the different ages, and the fact that the analyzed adipose tissue depot was not the same [70]. Regarding scWAT, *p27* tended to be upregulated in the overweight group and in the obese with type 2 DM patients, which agrees to that observed in the scWAT of DIO mice. Similarly, *CDK2* was also slightly increased in human scWAT in obesity which was also observed in the same depot of DIO mice. Together, these results suggest that *CDK2* and *p27* could have a critical role in WAT development, especially on scWAT.

Another interesting finding of the current study was the observation that high expression of *p27* and *CDK2* in scWAT was associated with bigger scWAT depot in humans. These positive associations were also observed in mice, pointing to a possible role in the expansion of the scWAT depot. Additionally, no association was found between age and the expression of *p27* and *CDK*2 in neither of the depots. However, this could be a consequence of the limited number of subjects in each age range within the cohort. In order to corroborate this result, a larger cohort with enough individuals representing each age range would be necessary. Human obesity is affected by several factors, such as environment, lifestyle, onset and duration of obesity and genetic background, while obesity can be induced in mice under controlled conditions avoiding the influence of most of these factors [71,72]. This could partly explain some inconsistencies between the data obtained in the human and mice studies. Several works have reported the difficulties of extrapolating or replicating the results gathered from mouse models in human [73,74], whereas other studies prove the validity of these models [75,76]. Besides, BMI was used in the human study to perform different correlation analyses as it is a well-established parameter to classify obesity [77], although when used without additional measurements, it is an insufficient marker of adiposity [78]. For this reason, correlation analyses were also performed using scWAT and vWAT areas measured by computed tomography (CT), a widely recognized technique for accurately assessing body fat distribution [79,80,81,82]. 

It has been shown that male and female mice have different susceptibility to develop obesity [83]. Furthermore, a recent study has shown differences in adipose tissue adaptability and metabolic health between aged obese female and male mice [49]. It was also reported that long term HFD-fed female mice presented larger gWAT and scWAT depots compared to male mice [84], which could be influenced by the function of the cell cycle regulators [51]. Our study in aged CT and DIO mice was performed only in females, and therefore further studies in aged male mice are needed to discern the possible differential regulation of the cell cycle regulators in obesity and aging between both sexes. In humans, differences on adipose tissue depots between men and women have also been described due to hormonal regulation, which affects its distribution, quantity and metabolic capacity [85]. There are sex differences in the adiposity accumulation beyond the well-known major deposition of fat in scWAT in females vs. vWAT in males [86]. However, the molecular mechanisms underlying these findings are still a matter of study. For this reason, our study includes the same number of men and women in the four groups. To characterize a potential sexual dimorphism in the regulation of *p27* and *CDK2* expression in WAT, the analysis was also performed separately for men and women, showing both men and women similar trends in *p27* and *CDK2* expression. However, the correlation analyses revealed that higher levels of *CDK2* in scWAT positively associated with triglycerides and BMI in men but not in women. Conversely, the significant positive association between *p27* and *CDK2* expression in scWAT and the size of this fat depot found in the whole cohort was not observed when the cohort was divided in men and women, probably because of the limited number of subjects within each group with body fat measurements by CT scans. Furthermore, the human cohort was formed by individuals within a wide range of age, making it difficult to establish associations between the expression of *p27* and *CDK2* and age. Finally, the studies were performed with whole adipose tissue, for both human and mice, which apart from mature adipocytes contains several cell types included in the stroma-vascular fraction (endothelial cells, preadipocytes, immune cells, among others), which could be an additional confounding factor in understanding the observed results. Therefore, it will be of interest to carry out future studies analyzing the expression of p27, cdk2 and associated cyclins both in isolated adipocytes and the stroma vascular fraction of the different depots of adipose tissue.

Taken together, the results obtained suggest an important transcriptional regulation of p27 and cdk2 in adipose tissue during aging and obesity. However, further studies are necessary to better characterize if these changes are the cause or consequence of these processes, as well as to discern the potential role of these cell cycle regulators within adipocyte metabolism, and the susceptibility to regulate obesity and aging.

## 4. Materials and Methods

### 4.1. Animal Models

The main study was carried out in 7-week-old female C57BL/6J mice obtained from Harlan Laboratories (Barcelona, Spain). Mice were kept under controlled conditions of temperature (21 ± 2 °C), light/dark cycles (12 h/12 h) and humidity (50 ± 10%). After 10 days of acclimation, mice were divided in the following experimental groups: a control group (CT), fed with standard laboratory diet and a DIO group fed with HFD. The standard diet provided 20% proteins, 67% carbohydrates, and 13% lipids (2014 diet, Harlan Teklad Global Diets, Harlan Laboratories, Indianapolis, IN, USA), and the HFD provided 45% fat, 20% protein and 35% carbohydrates (Research Diets, New Brunswick, IN, USA). Two-month-old mice were fed ad libitum with either of the two diets and were sacrificed at different time points, organizing the study in the following groups: young CT (2 months, *n* = 8), adult CT (6 months, *n* = 7), aged CT (18 months, *n* = 8) and aged DIO (18 months, *n* = 9). At the endpoint, body composition was measured by magnetic resonance technology (EchoMRI-100-700; Echo Medical Systems, Houston, TX, USA) in non-fasted mice. After overnight fasting, mice were sacrificed and the different adipose tissue depots (gWAT as a vWAT, scWAT and iBAT) were collected, weighed, and placed in liquid nitrogen before being stored at −80 °C. 

For the male mice study, C57BL/6J mice (7 weeks-old) were obtained from Harlan Laboratories and housed in the same conditions of temperature (light/dark cycles and humidity explained in the female study). Standard diet was the same than in the female study. HFD was provided by Research Diets with a distribution of macronutrients of 60% of kcal from fat, 20% from carbohydrates, and 20% from protein. After a 7 d acclimation period, C57BL/6J male mice were split into 2 groups: a control group (CT) that was fed a standard diet (n = *5*) and an HFD-fed group (DIO) (n = *5*). Animals were fed these diets ad libitum for 3 months. At the endpoint, scWAT depots were collected, weighed and placed in liquid nitrogen before being stored at −80 °C. 

All experiments were performed according to National Animal Care guidelines, and with the approval of the Ethics Committee for Animal Experimentation of the University of Navarra (protocol numbers: 113-15 and 047-15) in accordance with the EU Directive 2010/63/EU.

### 4.2. Human Samples

The study with human samples was carried out in vWAT and scWAT biopsies from the FATe cohort [87]. BMI was calculated during patient visits from heights and weights obtained with appropriate calibrated devices. This cohort included subjects who met the inclusion and exclusion criteria by BMI as: normal-weight (20–24.9 kg/m^2^, *n* = 10), overweight (25–29.9 kg/m^2^, n = 10), obesity (≥30 kg/m^2^, *n* = 20) and obesity with type 2 DM (≥30 kg/m^2^ and HbA1c ≥6.5%, *n* = 20). The study was approved by the Aragon Ethics Committee of Investigation (20/2014) with the informed consent of all the participants, and by the Research Ethics Committee of the University of Navarra (protocol 2019.153). For this study, 60 adult participants were selected from the cohort (50% women and 50% men in each group). Subjects were divided into 4 groups according to their BMI and the presence or not of type 2 DM. Additionally, vWAT and scWAT biopsies were obtained during elective surgeries and stored in the Biobank of the Aragon Health System at −80 °C in cryopreservation tubes until RNA extraction. 

### 4.3. Blood Analyses

Plasma determinations of glucose, triglycerides, cholesterol, insulin, and HbA1c were performed at the Clinical Biochemistry Department in the Miguel Servet University Hospital (Zaragoza, Spain) using state of the art analyzers. All analyses were in compliance with the requirements for quality and competence (ISO 15189:2012) for medical laboratories.

### 4.4. Determination of Body Fat Distribution and Content in Humans

As described elsewhere [87], the visceral and subcutaneous fat were measured by CT with an 8 mm single slice at the umbilical level. All CT examinations were acquired with the subject positioned supine in a 64 detector CT scanner (Aquilion 64 Toshiba Tokyo, Japan) and tube voltage set to 120 kVp with automatic tube current modulation and rotation time of 0.5 s. Acquired images were then transferred to a workstation and analyzed with the Vitrea CT Fat Measurement software (Vital Imaging Inc. The Netherlands). Selected fat densities ranged between −150 and −70 Hounsfield Units (HU) and the total subcutaneous and visceral fat areas were measured in cm^2^.

### 4.5. RT-PCR

RNA from mouse and human samples was extracted with TRIzol™ reagent (Invitrogen, ThermoFisher Scientific, Waltham, MA, USA), according to the manufacturer’s instructions. RNA quality and concentrations were measured by Nanodrop Spectrophotometer ND1000 (Nanodrop Technologies, Inc. Wilmington, NC, USA). RNA (1–5 µg) was then incubated with DNase I (RapidOut DNA Removal kit, Thermo Fisher Scientific) for 30 min at 37 °C and reverse transcribed to cDNA using the High-Capacity cDNA Reverse Transcription Kit (Applied Biosystems; Thermo Fisher Scientific) according to the manufacturer’s instructions in a Touch PCR system (C1000, BIO-RAD, Hercules, CA, USA). Real-time PCR was performed using the Touch Real-Time PCR System (CFX384, BIO-RAD). The expression of genes was determined using Power SYBR Green PCR Master Mix (BIO-RAD) or TaqMan master mix (Applied Biosystems). SYBR Green primers were obtained from published studies and tested with Primer-Blast software (National Center for Biotechnology Information, Bethesda, MD, USA; https://www.ncbi.nlm.nih.gov/tools/primer-blast). Primer sequences are shown in Table 4. *36b4* and *β-ACTIN* were used as housekeeping genes in the mouse and human study respectively. Relative expression of the specific genes was determined using the 2^−∆∆Ct^ method [88].

### 4.6. Statistical Analysis

The results are presented as mean ± SEM. For the statistical analysis of the results, GraphPad Prism 9.0 software was used (Graph-Pad Software, La Jolla, CA, USA). The comparison among the different groups was performed with one-way ANOVA or Kruskal–Wallis test followed by post hoc test for multiple groups-comparisons after testing the normality with Shapiro–Wilk tests. Student’s t test was used for comparisons between two groups. Correlation analyses were carried out by Pearson or Spearman correlation test for variables following parametric or non-parametric distribution respectively.

## Figures and Tables

**Table 1:** Correlation analyses between the weight of gWAT, scWAT or iBAT from Aged CT and Aged DIO female mice, and the mRNA expression of *p27*, *cdk2*, *ccna* or *ccne* in each depot.

	gWAT (g)		scWAT (g)		iBAT (g)	
	r	*p*	r	*p*	r	*p*
*p27*	0.15 ^a^	0.63	0.77 ^b^	<0.05	−0.21 ^b^	0.48
*cdk2*	0.09 ^a^	0.75	0.73 ^a^	<0.05	0.62 ^b^	<0.05
*ccna*	0.85 ^a^	<0.001	0.05 ^a^	0.88	0.69 ^a^	<0.05
*ccne*	−0.07 ^a^	0.81	0.09 ^a^	0.83	0.33 ^a^	0.25

r: Pearson’s (^a^) or Spearman’s (^b^) correlation coefficient. *p* < 0.05 is considered statistically significant (*n* = 10–14).

**Table 2:** Baseline characteristics and biochemical parameters of the different groups within the cohort.

	Normal-Weight	Overweight	Obesity	Obesity + DM
Sex (Men/Women)	5/5	5/5	10/10	10/10
Age (yr)	47.90 ± 10.56	53.89 ± 14.01	47.75 ± 10.86	56.05 ± 10.66
BMI (kg/m^2^)	22.81 ± 1.70	28.51 ± 0.87 **	36.58 ± 4.88 ***	37.43 ± 4.16 ***
vWAT area (cm^2^) ^a^	88.38 ± 56.38	121.50 ± 60.86	182.60 ± 113.80	326.90 ± 79.11 ***^$$^
scWAT area (cm^2^) ^a^	103.60 ± 62.82	208.40 ± 88.70	320.80 ± 126.90 **	347.50 ± 137.70 **
Glucose (mg/dl)	74.00 ± 5.03	77.20 ± 6.97	90.63 ± 17.70	135.00 ± 35.06 ***^$$$^
Triglycerides (mg/dl)	91.74 ± 27.47	108.40 ± 46.66	123.08 ± 49.41	122.50 ± 36.54
Cholesterol (mg/dl)	217.80 ± 28.98	215.40 ± 22.31	202.60 ± 38.71	176.50 ± 29.37 *
HDL-cholesterol (mg/dl)	58.00 ± 13.13	54.10 ± 12.20	48.58 ± 10.96	48.71 ± 12.07
LDL-cholesterol (mg/dl)	138.20 ± 22.29	139.1 ± 25.07	131.10 ± 36.92	91.43 ± 26.29 **^$$^
Insulin (mU/L)	4.23 ± 1.76	4.67 ± 1.95	5.94 ± 4.10	10.45 ± 6.13 **^$^
HbA1c (%)	5.32 ± 0.27	5.38 ± 0.31	5.43 ± 0.29	7.47 ± 1.59 ***^$$$^

Data are expressed in mean ± SD. * *p* < 0.05, ** *p* < 0.01, *** *p* < 0.001 vs. normal-weight. ^$^ *p* < 0.05, ^$$^ *p* < 0.05, ^$$$^ *p* < 0.05 vs. obesity. HbA1c; glycated hemoglobin. ^a^ Measured in 33 subjects (normal-weight, *n* = 5; overweight, *n* = 4; obesity, *n* = 14; obesity + DM, *n* = 10)

**Table 3:** Correlation analyses of *p27* and *CDK2* mRNA expression in human vWAT and scWAT with age, BMI, vWAT and scWAT area, serum glucose and triglycerides.

	vWAT				scWAT			
	*p27*		*CDK2*		*p27*		*CDK2*	
	r	*p*	r	*p*	r	*p*	r	*p*
Age (yr)	0.20	0.13	0.13	0.35	0.04	0.75	−0.09	0.54
BMI (kg/m^2^)	0.22	0.10	0.06	0.68	0.15	0.26	0.24	0.08
vWAT area (cm^2^)	0.09	0.62	0.09	0.60	0.05	0.78	0.11	0.56
scWAT area (cm^2^)	0.19	0.31	0.16	0.38	0.38	<0.05	0.44	<0.05
Glucose (mg/dL)	0.20	0.14	0.01	0.95	0.04	0.74	0.19	0.16
Triglycerides (mg/dL)	−0.08	0.57	−0.10	0.48	0.13	0.37	0.25	0.09

r: Spearman’s correlation coefficient. *p* < 0.05 is considered statistically significant.

**Table 4:** Sequences of primers used in the study.

Primer	Species	Sequence	Reference
*p27*	*Mus musculus*	Fw: CAGAATCATAAGCCCCTGGA	[89]
		Rv: TCTGACGAGTCAGGCATTTG	
*cdk2*	*Mus musculus*	Fw: CATTCCTCTTCCCCTCATCA	[90]
		Rv: GCAGCCCAGAAGAATTTCAG	
*ccna*	*Mus musculus*	Fw: CTTGGCTGCACCAACAGTAA	[91]
		Rv: ATGACTCAGGCCAGCTCTGT	
*ccne*	*Mus musculus*	Fw: CCTCCAAAGTTGCACCAGTT	[92]
		Rv: GGACGCACAGGTCTAGAAGC	
*p21*	*Mus musculus*	Fw: CTGTCTTGCACTCTGGTGTCTGA	[93]
		Rv: CCAATCTGCGCTTGGAGTGA	
*p57*	*Mus musculus*	Fw: GCCGGGTGATGAGCTGGGAA	[94]
		Rv: AGAGAGGCTGGTCCTTCAGC	
*Lep*	*Mus musculus*	Fw: CTCCATCTGCTGGCCTTCTC	[95]
		Rv: CATCCAGGCTCTCTGGCTTCT	
*36b4*	*Mus musculus*	Fw: CACTGGTCTAGGACCCGAGAAG	[96]
		Rv: GGTGCCTCTGGAGATTTTCG	
*p27*	*Homo sapiens*	Fw: TCTGAGGACACGCATTTGG	[97]
		Rv: TGTTCTGTTGGCTCTTTTGTTT	
*CDK2*	*Homo sapiens*	Hs01548894_m1	-
*Β-ACTIN*	*Homo sapiens*	Fw: CATCGAGCACGGCATCGTCA	[98]
		Rv: TAGCACAGCCTGGATAGCAAC	

## Data Availability

Not applicable.

## Supplementary Materials

The following are available online at https://www.mdpi.com/article/10.3390/ijms222111745/s1.
