# Supplementary material for: Regulation of p27 and Cdk2 Expression in Different Adipose Tissue Depots in Aging and Obesity
Source: Int J Mol Sci. 2021 Oct 29;22(21):11745. doi: 10.3390/ijms222111745 (PMC8584112; doi:10.3390/ijms222111745)
Supplement: Supplementary file 1 [file ijms-22-11745-s001.zip › ijms-1398859-supplementary.pdf]

Supplementary Material

Regulation of p27 and Cdk2 Expression in Different Adipose Tissue Depots in Aging and Obesity

Ignacio Colón-Mesa[1], Marta Fernández-Galilea[1,2], Neira Sáinz[1], Marta Lopez-Yus[3], Jose M. Artigas[4], José Miguel Arbonés-Mainar[3,5], Elisa Félix-Soriano[1], Xavier Escoté[1,6,7,*,#] and María Jesús Moreno-Aliaga[1,2,5,#]

[1] Center for Nutrition Research and Department of Nutrition, Food Science and Physiology, University of Navarra, 31008 Pamplona, Spain; icolon@alumni.unav.es (I.C.-M.); mfgalilea@unav.es (M.F.-G.); nsainz@unav.es (N.S.); efelix@alumni.unav.es (E.F.-S.); mjmoreno@unav.es (M.J.M.-A.)
[2] Navarra Institute for Health Research (IdiSNA), 31008 Pamplona, Spain
[3] Adipocyte and Fat Biology Laboratory (AdipoFat), Unidad de Investigación Traslacional, Instituto Aragonés de Ciencias de la Salud (IACS), Instituto de Investigación Sanitaria (IIS) Aragón, Hospital Universitario Miguel Servet, 50009 Zaragoza, Spain; martalyus@gmail.com (M.L.-Y.); jmarbones.iacs@aragon.es (J.M.A.-M.)
[4] Radiology Department, Hospital Universitario Miguel Servet, 50009 Zaragoza, Spain; jmartigasm@gmail.com (J.M.A)
[5] CIBERobn Physiopathology of Obesity and Nutrition, Carlos III Health Institute, 28029 Madrid, Spain
[6] Department of Biochemistry and Biotechnology, Universitat Rovira i Virgili, Campus Sescelades, 43007 Tarragona, Spain
[7] Eurecat, Technology Centre of Catalunya, Nutrition and Health Unit, 43204 Reus, Spain
* Correspondence: xavier.escote@eurecat.org; Tel.: +34-977-300-805
† These authors share the senior authorship.

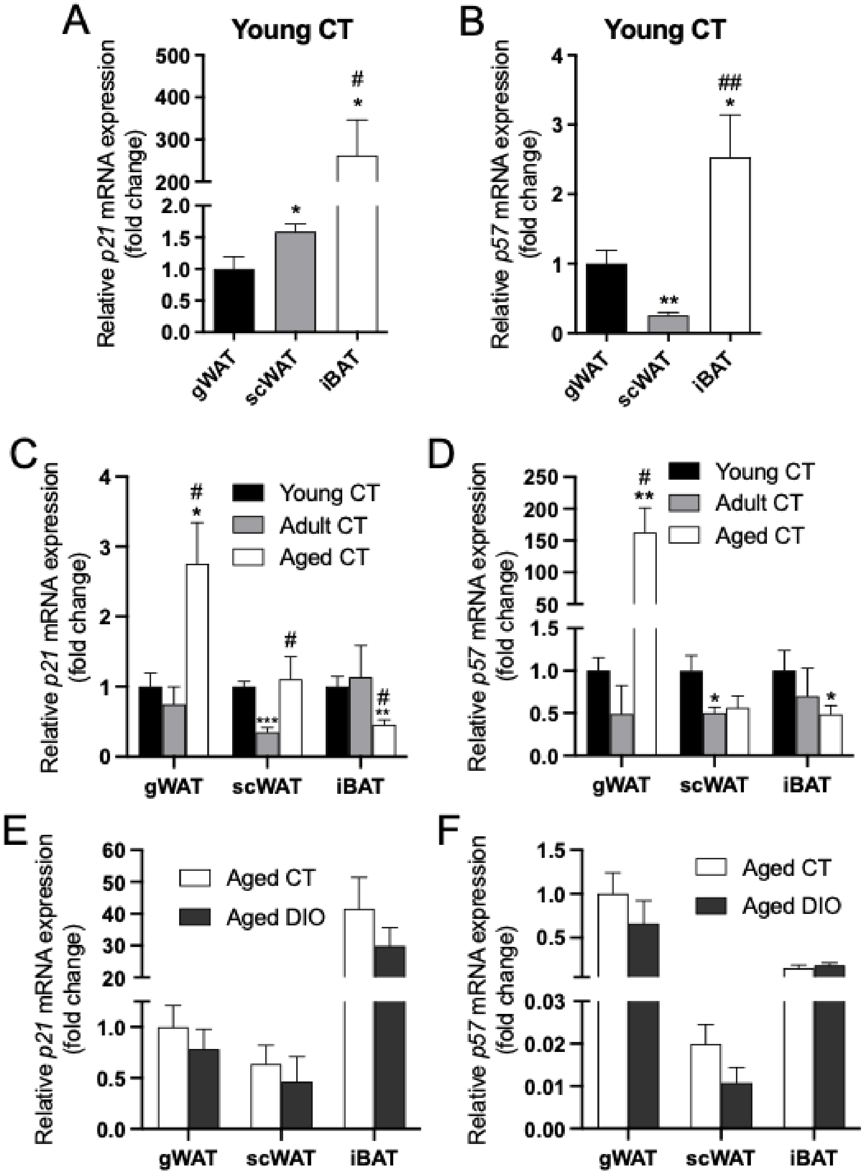

Supplemental Figure S1. **A, B:** Differential adipose tissue depot expression of *p21* (A) and *p57* (B), between gWAT, scWAT and iBAT in young CT mice (2-month-old). Data (mean ± SEM) are expressed as fold change of gWAT, considered as 1. *p<0.05, **p<0.01 vs. gWAT; #p<0.05, ##p<0.01, vs. scWAT. **C, D:** Changes induced by aging on the mRNA expression of *p21* (C) and *p57* (D) in gWAT, scWAT and iBAT from young, adult and aged (2-, 6- and 18-month-old) CT mice. Data (mean ± SEM) are expressed as fold change of Young CT, considered as 1. *p<0.05, **p<0.01, ***p<0.001 *vs.* Young CT; #p<0.05 *vs.* Adult CT. **E, F:** Effect of diet-induced obesity (DIO) on the expression of *p21* (E) and *p57* (F), in gWAT, scWAT and iBAT from aged (18-month-old mice). Data (mean ± SEM) are expressed as fold change of gWAT from Aged CT mice, considered as 1. (*n*=5-9).

Supplemental Table S1. Correlation analyses of *p27*, *cdk2*, *ccna* and *ccne* mRNA expression with *leptin* mRNA expression in scWAT from Aged CT and Aged DIO female mice.

	**scWAT**							
	p27		***cdk2***		***ccna***		***ccne***	
	r	*p*	r	*p*	r	*p*	r	*p*
Lep	0.65[b]	0.09	**0.77**[b]	**<0.05**	-0.12[a]	0.75	-0.09[a]	0.84

r: Pearson's ([a]) or Spearman's ([b]) correlation coefficient. p<0.05 is considered statistically significant.

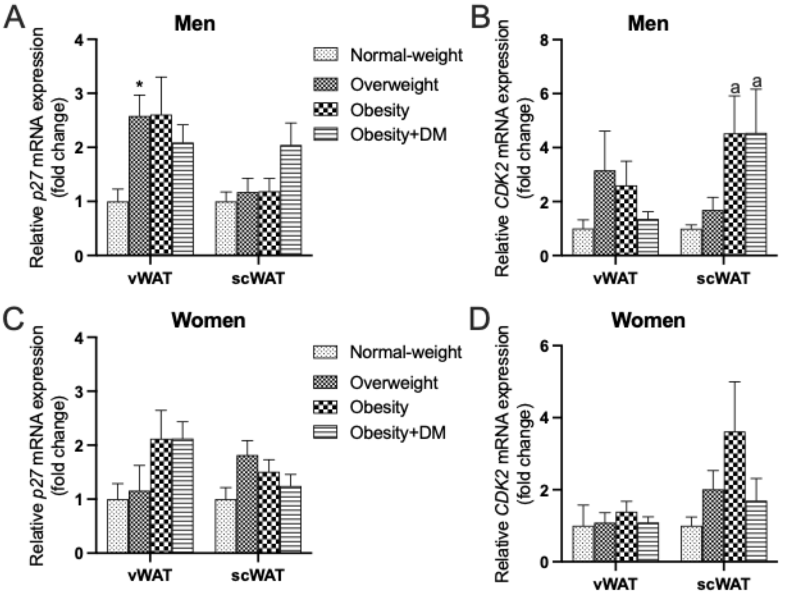

Supplemental Figure S2. Effects of overweight and obesity with or without type 2 DM on the mRNA expression of *p27* (A, C) and *CDK2* (B, D) in vWAT and scWAT from men (A, B) and women (C, D) Data (mean ± SEM) are expressed as fold change of Normal-weight, considered as 1. *p<0.05, [a]p=0.06 vs. Normal-weight.

Supplemental Table S2. Correlation analyses of *p27* and *CDK2* mRNA expression in vWAT and scWAT from men with age, BMI, vWAT and scWAT area, serum glucose and triglycerides.

	vWAT				*scWAT*			
	p27		*CDK2*		*p27*		*CDK2*	
	r	*p*	r	*p*	r	*p*	r	*p*
Age (yr)	0.24	0.22	0.15	0.42	0.11	0.58	-0.20	0.32
BMI (kg/m²)	0.17	0.37	-0.01	0.75	0.22	0.26	0.49	<0.05
vWAT area (cm²)[a]	0.34	0.23	0.07	0.81	0.28	0.33	0.10	0.75
scWAT area (cm²)[a]	-0.01	0.99	-0.09	0.76	0.50	0.07	0.51	0.08
Glucose (mg/dL)	0.08	0.70	-0.06	0.76	0.10	0.59	0.29	0.16
Triglycerides (mg/dL)	-0.14	0.51	-0.09	0.65	0.09	0.68	0.46	<0.05

r: Spearman's correlation coefficient. p<0.05 is considered statistically significant. [a]Correlation analysis performed with 15 subjects (Normal-weight, *n*=2; Overweight, *n*=1; Obesity, *n*=7; Obesity + DM, *n*=5).

Supplemental Table S3. Correlation analyses of *p27* and *CDK2* mRNA expression in vWAT and scWAT from women with age, BMI, vWAT and scWAT area, serum glucose and triglycerides.

	vWAT				*scWAT*			
	p27		*CDK2*		*p27*		*CDK2*	
	r	*p*	r	*p*	r	*p*	r	*p*
Age (yr)	0.19	0.33	0.16	0.42	-0.07	0.71	0.02	0.92
BMI (kg/m²)	0.30	0.13	0.24	0.23	-0.01	0.95	-0.01	0.98
vWAT area (cm²)[a]	0.02	0.93	0.12	0.65	-0.23	0.37	0.06	0.82
scWAT area (cm²)[a]	0.37	0.15	0.54	<0.05	0.22	0.40	0.33	0.21
Glucose (mg/dL)	0.25	0.21	0.13	0.51	-0.10	0.60	0.08	0.70
Triglycerides (mg/dL)	0.01	0.98	-0.11	0.61	0.19	0.36	-0.04	0.85

r: Spearman's correlation coefficient. p<0.05 is considered statistically significant. [a]Correlation analysis performed with 18 subjects (Normal-weight, *n*=3; Overweight, *n*=3; Obesity, *n*=7; Obesity + DM, *n*=5).
